# Translational medicine as a new clinical tool and application which improves metabolic diseases: perspectives from 2012 Sino-American symposium on clinical and translational medicine

**DOI:** 10.1186/2001-1326-3-2

**Published:** 2014-02-10

**Authors:** Lin Shi, Elena López Villar, Chengshui Chen

**Affiliations:** 1 Department of Pulmonary Medicine, The First Affiliated Hospital, Wenzhou Medical University, Wenzhou 325000, China; 2 Hospital Universitario Infantil Niño Jesús, Madrid, Spain

**Keywords:** Metabolic diseases, Clinical translational medicine, Hepatotoxicity, Colorectal cancer, Obesity, Type 2 diabetes mellitus, microRNAs

## Abstract

Because of the economic growth and changes in lifestyle, metabolic diseases have become a major public health problem, which impose heavy economic burdens on individuals, families and health systems. However, its precise mediators and mechanisms remain to be fully understood. Clinical translational medicine (CTM) is an emerging area comprising multidisciplinary research from basic science to medical applications and as a new tool to improve human health by reducing disease incidence, morbidity and mortality. It can bridge knowledge of metabolic diseases processes, gained by in vitro and experimental animal models, with the disease pathways found in humans, further to identify their susceptibility genes and enable patients to achieve personalized medicament treatment. Thus, we have the reasons to believe that CTM will play even more roles in the development of new diagnostics, therapies, healthcare, and policies and the Sino-American Symposium on Clinical and Translational Medicine (SAS-CTM) will become a more and more important platform for exchanging ideas on clinical and translational research and entails a close collaboration among hospital, academia and industry.

## Introduction

Lifestyle changes, such as decreased physical activities, increased excessive caloric intake, or sedentary behavior, all contribute to the progression of metabolic diseases, such as diabetes, obesity, or atherosclerosis, at an alarming rate and have become a major public health problem [1,2]. The potential correlation between metabolic diseases and cancer attracts more attention from scientific societies. CTM has been suggested as an emerging area comprising multidisciplinary research transforms and scientific discoveries and as a new tool to improve human health by reducing disease incidence, morbidity and mortality. Indeed, to improve health, findings from basic research studies must be translated into practical applications. Translational research transforms scientific discoveries found in the laboratory into ways to prevent, diagnosis or treat disease [3]. However, precise mediators and mechanisms need to be further clarified [4]. There is still great variability to the response to treatment of metabolic diseases, due to a number of factors, e.g. genetics, environment, and behavior [5]. It is also important for clinical and translational scientists to consider how to translate the individual therapy into public health models for disease prevention or treatment in a low-resource setting. The SAS-CTM provides one of the highest and most influential platforms to obtain the latest development and explore new opportunities in international collaborations for metabolic diseases.

## Review

### Translational research in hepatotoxicity

Hepatotoxicity (hepatic toxicity) implies chemical-driven liver damage. The liver has a central function in transforming and clearing chemicals but it is susceptible to the toxicity from these agents. when taken Certain medicinal agents in overdoses, may injure the organ. Drug-induced liver injury (DILI) limits the development and application of many therapeutic compounds and presents major challenges to the pharmaceutical industry and clinical medicine [6]. It is a major safety concern in drug development and clinical pharmacotherapy. The most problematic type of DILI is “idiosyncratic”. This means that only a very small fraction of treated patients are susceptible to DILI, e.g. Carbamazepine (CBZ) a widely used anti-epileptic agent, is generally well tolerated-only a small number of patients prescribed CBZ develop severe hepatitis [7]. Idiosyncratic DILI is a complex disorder that is difficult to predict, diagnose and treat [8,9]. Underlying metabolic disorders have been suggested as being a predisposing factor for DILI. Prof. Paul B. Watkins, University of North Carolina-Chapel Hill, NC, explained the role of CTM in DILI with a special statement on *Understanding hepatotoxicity – From patients to mice to computer.* In addition to genetic studies of patients who have experienced DILI from US Drug-Induced Liver Injury Network (DILIN), they are partnering with Cellular Dynamics International to reprogram induced pleuripotent stem cells from DILIN subjects and are also co-operating with the Shanghai Centers for Disease Control to prospectively collect a variety of bio-specimens as part of a larger biomarker discovery initiative.

DILI remains the major adverse drug event that leads to termination of clinical development programs and regulatory actions including failure for marketing approval, restricted indications, and withdrawal from the marketplace. There are more and more preclinical models for its study. Reliable preclinical testing will probably not be developed until there is greater understanding of the mechanisms underlying DILI. Prof. Watkins *et al.* are using panels of inbred mice to mimic patient population genetic diversity and to identify genes and pathways that may underlie DILI susceptibility in patients. As a result, the inbred mouse panel approach successfully identified an unsuspected genetic factor underlying susceptibility to acetaminophen (paracetamol) hepatotoxicity in humans. Finally, Prof. Watkins demonstrated that they have established the DILIsim Initiative, which is a public-private partnership that is building a computer model to synthesize the rapidly accumulating data with the goal of predicting DILI liability in drug candidates.

### Translational research in human colorectal cancer

Colorectal cancer is an important cause of mortality in our society. The progression of this disease from the normal colonic-epithelium to the malignant-phenotype is occurs with numerous genetic and epigenetic alterations. Compelling experimental and omics researches, indicates that nutrition and diet are important keys in the modulation of colorectal cancer [10]. In order to sustain higher proliferative rates and resist some cell death signals, tumor cells exhibit distinct metabolic phenotypes to alter the flux along key metabolic pathways, such as glycolysis and glutaminolysis [10,11]. When used as a translational research tool, metabolomics is the upcoming new science in the omics field with the potential to further increment our knowledge of cancer biology [12]. Cancer metabolomics research aims at evaluating and predicting pathophysiological changes of cancer patients by investigating metabolic signatures in body fluids or tissues, which are influenced by genetics, epigenetics, environmental exposures, diet, and behavior. Prof. Wei Jia, University of North Carolina at Greensboro, described their studies with mass spectrometry based on metabolomic profiling of serum, urine and tissue samples from colorectal cancer (CRC) patients entitled *“Metabolic profiling of human colorectal cancer: A Top-down Approach to Translational Cancer Research”.* A particular advantage of metabolomics is that it represents a top-down tactic in that all of the molecules detected are interrogated, providing a global picture of dynamic metabolic changes involving key markers and pathways those were not already associated with carcinogenesis.

Metabolomics is a powerful tool for biomarker discovery and the investigation of pathological processes, phenotypic variations, and treatment effects. The metabolic profile of CRC involves several significantly altered metabolic pathways, including increased glycolysis and an impaired tricarboxylic acid cycle, dysregulations of glutaminolysis and down-regulated urea cycle. In addition, dysregulation of amino acids and nucleotides, carnitine family, choline family, and gut microbial co-metabolites were also observed. Prof. Jia demonstrated their experimental results and emphasized the potential for the metabolomic approach for having a multitude of use in oncology, including the early detection and diagnosis of cancer and as both a predictive and pharmacodynamic marker of therapeutic effect.

### Translational research in obesity

Obesity, worldwide, is becoming a major problem with an increased risk of several common diseases including diabetes, cardiovascular disease, and cancer [13,14]. It is generally accepted that hypoxia is related to sleep apnea in obesity. This concept has changed since the finding of hypoxia response in adipose tissue of obese mice in 2005. Adipose tissue is a key endocrine organ as it releases multiple bioactive substances, known as adipose-derived secreted factors or adipokines, that have pro-inflammatory or anti-inflammatory activities [15]. Recent studies consistently support a hypoxia response in the adipose tissue in obese animals. The observations have led to the formation of an exciting concept: adipose tissue hypoxia (ATH), in the understanding of major disorders associated with obesity [16]. Prof. Jianping Ye, Louisiana State University, addressed the *Hypoxia in Obesity-From Bench to Bedside.* The ATH provides a unified answer to all of the pathological changes in the adipose tissue under obesity, such as chronic inflammation, endoplasmic reticulum stress, leptin expression, adiponectin reduction, adipocyte death and elevated lipolysis.

The ATH suggests that capillary dysfunction occurs during expansion of adipose tissue, and leads to reduction in adipose blood supply, which is responsible for hypoxia in obesity. The adipose tissue dysfunction is a result of local vascular failure. In addition, it stimulates the inflammatory response in favor of the adipose tissue remodeling and energy expenditure to fight against obesity. These new insights into the ATH suggest that the hypoxia response also has a beneficial effect in the body. Prof. Ye emphasized that sleep apnea is a protective mechanism in feedback to obesity by using the hypoxia response to trigger the onset of multiple protection mechanisms in the body.

Many millions of years of evolution have passed from ape to human. We found that humans began a slow but gradual manifestation of the state of obesity. But what are the reasons that lead to obesity pathogenesis? Is it the result of evolution? Or the unhealthy life style? With these questions, Prof. Guang Ning, The Vice President of Ruijin Hospital Affiliated Shanghai JiaoTong University School of Medicine, retrospected the obesity prevalence and its relationship with economic development and lifestyle transition in China entitled “*Translational Medicine in China: Improving Public Health”* that consider obesity as a complex disease, resulting from both external and internal causes. The external causes usually mean environmental factors including socio-economy, life style, nutrition and others. The internal causes refer to genetic factors. Furthermore, the prevalence of diabetes gradually increased with the development of obesity. Prof. Ning suggested that the translation of this view into clinical setting, will provide clinical evidence for the interaction between obesity and diabetes.

### Translational research in type 2 diabetes mellitus

The incidence of type 2 diabetes mellitus (T2DM) is increasing at an alarming rate, in part due to the dramatic rise in obesity in developed countries, as T2DM is a comorbidity frequently seen in obese subjects. Because not all obese patients develop T2DM and not all subjects suffering from T2DM are obese or overweight [17], it is of vast interest to understand the mechanisms underlying the association between these two entities in order to predict which patients are at higher risk for developing this disease, and possibly for the development of preventative therapies.

Because of the economic growth and changes in lifestyle, diabetes has become an important public health problem. *e.g.* a national study from 2007 to 2008 showed that the age-standardized prevalences of diabetes and prediabetes were 9.7% and 15.5%, respectively, accounting for 92.4 million adults with diabetes and 148.2 million adults with prediabetes [1]. Compared with 1979, the prevalence of diabetes increased by 8 times in 2008. Alarmingly, the rapid rise in obesity and T2DM in all age groups might result in a substantial increase in prevalence of diabetes-related cognitive dysfunction [17]. T2DM is influenced by the environmental factors and genetic factors [18]. According to the heterogeneity in environmental factors as well as genetic background, the susceptibility of T2DM in different ethnic populations is totally different, with low susceptibility in Caucasians, while little susceptibility in Chinese Hans. Prof. Weiping Jia, President of Shanghai Jiao Tong University Affiliated Sixth People’s Hospital, emphasized the importance of *Susceptible genes of type 2 diabetes and their disease predictive power in Chinese population.*

The recent success of genetics has identified 50 susceptible genes of T2DM in Caucasians by biology candidate study, linkage study and also genome-wide association study, among which some loci such as KCNJ11, TCF7L2, TCF2 genes have been replicated successfully in our Chinese Han population with the odds ratio ranging from 1.14-1.31. Besides, by replicating the susceptible genes specific for the East Asian, 6 loci showed significance in our Chinese samples, with KCNQ1 showing the strongest association with T2DM [19]. However, although nearly 50 susceptible genes for T2DM were identified so far, the prediction effects were still limited. Thus, Prof. Jia suggested that the utility of both genetic and environmental factors will be worthwhile exploring in the disease prediction.

In addition, genetic factors involved in drug absorption, distribution, metabolism and target attribute to differences between patients and affect clinical anti-diabetic treatments. The identification of genetic markers related to drug reaction can help physicians with the decisions of (a) drug selection, (b) dose titration, (c) treatment duration, and (d) avoidance of advert drug reactions. These advances in understanding the genetics of T2DM will lead to the development of new therapeutic and preventive strategies and individualized medicine. Associate Prof. Cheng Hu, Shanghai Jiao Tong University Affiliated Sixth People’s Hospital, focused on the effects of susceptibility genes for T2DM on anti-drugs’ efficacy and addressed *Pharmacogenomics in type 2 diabetes management: towards personalized medicine.* It had been already suggested that effects of some genetic variants on the response to repaglinide and rosiglitazone affect either its pharmacokinetics or pharmacodynamics. In spite of all these advances in the field of pharmacodynamics of T2DM, the pace of clinical application of these findings is rather slow. Associate Prof. Hu advised that more research, especially randomized clinical trials into practical utility, should be conducted.

### Translational research in microRNA

MicroRNAs (MiRNAs) are a class of small non-coding RNAs that negatively regulate gene expression at the post-transcriptional level by binding to homologous regions in the target mRNAs blocking translation and/or inducing mRNAs degradation [20]. Dysregulated expression of miRNAs in various tissues has been associated with a variety of diseases, including cancer [21,22]. For example, one of the first characterized miRNA families relevant in carcinogenesis was the miR-17-92 cluster which is involved in immune system regulation and is up-regulated in lung cancer [23]. The levels of miRNAs in serum are stable, reproducible, and consistent among individuals of the same species. Prof. Chenyu Zhang, Life Science, Nanjing University, introduced their early experimental work about sequencing all serum miRNAs of healthy Chinese subjects and identifying specific expression patterns of serum miRNAs for lung cancer, colorectal cancer and diabetes with a special statement on *Circulating MicroRNA, Secreted MicroRNA and Exogenous Plant MicroRNA.* Through these analyses, we conclude that serum miRNAs can serve as potential biomarkers for the detection of various cancers and other diseases.

Here, Prof. Zhang also report that secreted miRNAs can serve as novel signaling molecules mediating intercellular communication. They found that THP-1 cells can actively and selectively package miR-150 into microvesicles and secrete them into circulation. Microvesicles then can deliver miR-150 into human microvascular endothelial cells, resulting in suppression of c-Myb (a known target gene for miR-150) in recipient cells and enhanced migration capacity. These results demonstrate that cells actively secreted miRNAs and deliver them into specific recipient cells where the exogenous miRNAs can regulate target gene expression and recipient cell function.

### A grand challenge about multiple metabolic controls

Metabolic diseases and their complications are imposing heavy economic burdens on individuals, families, health systems and countries including China [24]. Take diabetes as an example: the clinical characteristics of T2DM appear several years after the onset of this process during which time the person is asymptomatic. Once T2DM clearly appears, it is not possible to reverse the disease progression. Despite this, it is possible and of utmost importance to properly control T2DM through diet and life style (e.g. exercise), following clinical advise. Thus, early detection of, or even more importantly, the possibility to avoid, T2DM onset would be of extreme benefit. Moreover, in diabetic patients, the prevalence of diabetic retinopathy was 16.9%. The prevalence of albuminuria and peripheral vascular disease was 25.7% and nearly 15% [25,26]. All these chronic complications may cause serious consequences, including blindness, renal dysfunction, strokes, myocardial infarction and amputation. Since 2003, with the support of Department of Disease Control of the Chinese Ministry of Health, Chinese Diabetes Society of the Chinese Medical Association published the Guideline for Diabetes Prevention and Treatment in Chinese. The guideline has been revised twice according to the evidence-based clinical trials. Prof. Yuqian Bao, Shanghai Jiao Tong University Affiliated Sixth People’s Hospital, described a Hospital-Community Diabetes Integrated Management entitled “*A grand challenge about multiple metabolic control: health care delivery from tertiary level hospital to primary care health center*” to increase “three rates” which include glucose control rate, screening rate of chronic complication and awareness rate of diabetes knowledge in Shanghai communities.

The Hospital-Community Diabetes Integrated Management established collaboration between urban hospitals (tertiary hospital) and community health services. The hospital and community health services have their own responsibilities. Tertiary hospital was in charge of training, establishing management guideline and providing a referral platform. The community health services center was in charge of organizing a management team and medical records, implementing the management according to the guideline and starting dual referral. Prof. Bao showed that up till now nearly 9,000 high risk individuals completed diabetes screening in 3 communities. And more than 5,000 medical records were managed by the community health services. The “three rates” improved significantly as consequence.

## Conclusions

**Figure 1:**
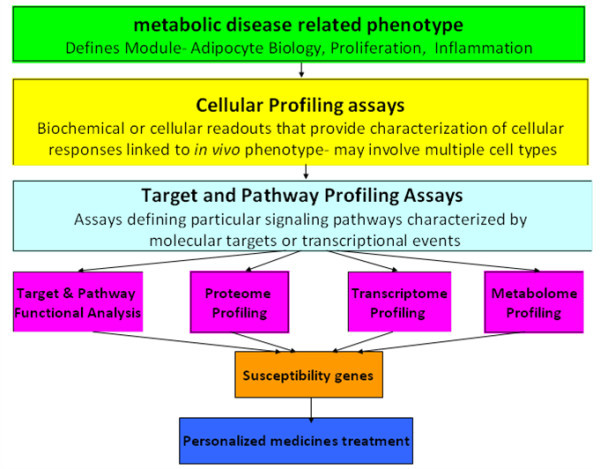
**CTM as a new clinical tool and application of personalized medicament treatment.** CTM focus on the metabolic disease related phenotype, then explores particular signaling pathway characterized by molecular targets or transcriptional events through biochemical or cellular readouts of cellular profiling assays, further to identify their susceptibility genes and enable patients to achieve a personalized medicines treatment.

In conclusion, metabolic diseases, a public health problem, the medical management of them has become increasingly complex, and its complications remain a great burden to individual patients and the larger society. Various mediators and mechanisms linking to metabolic diseases have been postulated. Modern health care is currently and globally undergoing a big revolution, where innovative therapies and novel technology advancements are having profound impacts on improving patient care and managing costs. One of the cornerstone with health care with great impact on metabolic diseases is the increasing number of personalized medicines being introduced into market. Much clinical interest lies in drug metabolism and the opportunity to improve prescribing efficacy and safety. CTM as a new clinical tool and application of clinical medicine and public health that faces great challenges and opportunities. It can bridge knowledge of metabolic diseases processes, gained by in vitro and experimental animal models, with the disease pathways found in humans, further to identify their susceptibility genes and enable patients to achieve personalized medicament treatment (Figure 1). In this way we have reason to believe that CTM will play even more roles in novel diagnostics/prognostics and therapies for clinical use, post-genomic knowledge and experience, and/or new disciplines that reflect additional levels of complexity. And SAS-CTM will become a more and more important platform for exchanging ideas on clinical and translational research and meet the demands of maintaining or expanding the biomedical workforce and education programs that attract and retain young people in translational and biomedical sciences.

## Abbreviations

ATH: Adipose tissue hypoxia; CBZ: Carbamazepine; CRC: Colorectal cancer; CTM: Clinical translational medicine; DILI: Drug-induced liver injury; DILIN: Drug- induced liver injury network; SAS: Sino-American Symposium; T2DM: Type 2 diabetes mellitus.

## Competing interests

The authors confirm that there are no conflicts of interest.

## Authors’ contributions

Authors LS, EL and CSC, carried out “Translational Medicine as a new clinical tool and application which improves metabolic diseases: Perspectives from 2012 Sino-American Symposium on Clinical and Translational Medicine” studies for this review, in order to develop future Clinical Translational Medicine research studies and publish this article. All authors read and approved the final manuscript.

## Authors’ information

LS and CSC are Dr. from The First Affiliated Hospital, Wenzhou Medical University (Wenzhou, China), EL is Dr. from SNS (Spanish Health System).
